# Sociodemographic characteristics of patients diagnosed with bipolar disorder manic episode hospitalized in a psychiatry clinic

**DOI:** 10.1192/j.eurpsy.2023.1463

**Published:** 2023-07-19

**Authors:** I. Ilter

**Affiliations:** psychiatry, hitit university, çorum, Türkiye

## Abstract

**Introduction:**

Bipolar disorder is a chronic mental illness that progresses with recurrent episodes and can cause serious loss of functionality.Sociodemographic characteristics have importance on the course of the disease.

**Objectives:**

In this study,sociodemographic data such as age,gender,educational status,job,and family history of patients with bipolar disorder manic episode who were followed up and treated in the psychiatry ward of our hospital were recorded.

**Methods:**

Our study included 50 patients diagnosed with bipolar disorder manic episode according to DSM-5 in the psychiatry service of Atatür kUniversity Hospital and 50 healthy volunteers who had not applied to psychiatry before. A 16-item sociodemographic chart in which age,gender,marital status,lifestyle,number of marriages, number of children,years of education,job,monthly income,maternal and paternal survival status, familyhistory of psychiatric illness,family history of bipolar disorder,smoking,alcohol and substance use were questioned.The data form was applied to the patients and healty volunteers.

**Results:**

After the statistical analysis between the groups, a significant difference was found in terms of lifestyle(p<0.001),job status(p=0.01), family history(p<0.01) and substance use history(p=0.04).

**Conclusions:**

The rate of psychiatric illness in the families of individuals diagnosed with bipolar disorder increases significantly compared to the normal population,and the data of our study are in line with the literature in this respect.It is known that conditions such as lower employment and interruption of working life are common in patients with bipolardisorder due to diseases such as loss of functionality and pharmacotherapy side effects.In our study,similar to the literature,the employment status was found to be significantly lower in the BD group compared to the healthy controls.In our study,no significant difference was found between the groups in terms of smoking and alcohol use,while other substance use was found to be significantly higher in the bipolardisorder group.Since factors such as sociocultural structure,education,geographical conditions,religious belief may affect smoking,alcohol and substance use;multicenter studies with larger samples are needed in order to more clearly reveal the rates associated with bipolar disorder and other psychiatric conditions.

**Disclosure of Interest:**

None Declared

